# Retrospective classification of veterinary forensic cases from the archives of the Italian Istituti Zooprofilattici Sperimentali (2013–2023)

**DOI:** 10.3389/fvets.2026.1856464

**Published:** 2026-06-22

**Authors:** Daniela Averaimo, Alessandra Scagliarini, Pietro Badagliacca, Stefania Salucci, Lucio Marino, Massimiliano Paoletti, Antonio Cocco, Elga E. Tieri, Maria Chiara Cantelmi, Sabrina V. P. Defourny, Angelo Peli, Antonio Petrini

**Affiliations:** 1 Istituto Zooprofilattico Sperimentale Abruzzo e Molise (IZSAM), Teramo, Italy; 2 Department of Medical and Surgical Sciences, Alma Mater Studiorum-Università di Bologna, Bologna, Italy; 3 Department for Life Quality Studies, Alma Mater Studiorum-Università di Bologna, Bologna, Italy

**Keywords:** categories, diagnosis labels, forensic necropsy, post-mortem reports, veterinary forensic medicine (VFM)

## Abstract

**Introduction:**

Definitive guidelines for the classification of cases in Veterinary Forensic Medicine (VFM) are currently unavailable. An attempt to classify lesions observed in forensic necropsies was proposed in 2022 following the codes of the ICD-11. The archives of the Istituti Zooprofilattici Sperimentali (IIZZSS) are structured to identify forensic pathology cases using pre-coded information only on wild animals' mortality and suspected animal poisonings at the time of acceptance. In contrast, other forensic documentation sources, including non-pre-coded necropsy diagnoses, require interpretation based on laboratory test results and accompanying expert opinions.

**Methods:**

This study proposes the classification of previously unclassified VFM cases through a three-step approach: (1) data analysis, (2) review and classification of expert opinions by assigning labels corresponding to the main forensic topics, and (3) organization of diagnoses into categories and subcategories. Cases recorded in the database of the Istituto Zooprofilattico Sperimentale dell'Abruzzo e del Molise between 2013 and 2023 were retrospectively evaluated.

**Results:**

A total of 1,221 non-coded cases with an expert opinion were identified. Among these, 405 cases (33.16%) were classified as potential Veterinary Forensic Cases and categorized into five macro-categories and 19 subcategories. The main cause of mortality differed according to animal type: injury was the most frequent cause in pets, attack in livestock, and unlawful killing in wildlife.

**Discussion:**

This classification approach enabled the identification and organization of previously unclassified VFM cases within the institutional database. Poisoning cases and wild animal deaths caused by vehicular collisions were excluded as they were already pre-classified. Therefore, the reported percentages refer only to this subset of cases and do not reflect the overall mortality rates in the region.

## 1 Introduction

Veterinary Forensic Science (VFS) is a specialized veterinary field aimed at providing scientific and technical support to authorities in order to bring perpetrators of animal-related crimes to justice (1). A key aspect of investigations by authorities is determining whether the criminal act was intentional, and therefore malicious, or the result of negligence. In the Italian legal system, there is a difference in the attribution of criminal responsibility between guilt, considered as an action without a deliberate intention to harm, and malice, where the offender acts with the intention of harming and is aware of the consequences of his action. The forensic necropsy plays a crucial role in this discrimination during investigations (2). Forensic veterinary pathologists are often asked to confirm whether injuries suspected of having been inflicted can indeed be confirmed as such, and how the injury may have been caused (3).

Like its human counterpart, VFS emphasizes objectivity and the need for clear documentation of events and findings (2). However, unlike human forensic medicine, VFS is not yet fully developed. Prior to 2008, peer-reviewed literature on veterinary forensic pathology in domestic animals was scarce. Since then, peer-reviewed literature on Veterinary Forensic Medicine (VFM) has increased exponentially (4). While numerous publications on forensic investigations of domestic animals and wildlife have emerged in recent years (5), there are still no definitive guidelines for classifying VFM cases. Although there is sometimes some confusion regarding the term “animal abuse,” as it encompasses various circumstances, and other terms such as “maltreatment” and “animal cruelty” are widely used, this confusion can be avoided by applying terms derived from the medical profession regarding “child abuse”. These include: physical abuse, sexual abuse, emotional abuse and neglect (6). These terms are commonly used by forensic pathologists. Despite this, VFM still lacks standardized guidelines for classifying anatomic-pathological injuries and cases falling under its domain, which are necessary to facilitate investigations and define responsibility. In 2022, Marchetti and colleagues (7) attempted to apply the ICD-11 codes used in human medicine to classify forensic veterinary pathology cases. This approach proved highly valuable for its clarity and simplicity in data processing. Specifically, Section 22, “Injury, poisoning, or certain other consequences of external causes”, was used to record the main immediate cause of death, defined by the WHO as “the final event in the causal sequence that occurred closest to the time of death”. While, to specify the event that triggered the chain of events leading to death (underlying cause of death), on which the determination of the manner of death is based, was used the Section 23, “External causes of morbidity or mortality” (7).

In Italy, the archives of the *Istituti Zooprofilattici Sperimentali* (IIZZSS), a network of public-law health institution with managerial and administrative autonomy, operating within the National Health Service, under supervision of the Ministry of Health) (8) contain many forensic pathology cases, structured for data extraction and analysis. When an “object of activity” is used to encode the samples before the analyses, such collections can be used to draft technical-scientific reports. An alternative way of data analysis from these archives is their classification in the aftermath by selective filters. Necropsies requiring interpretation of laboratory results (genetic, toxicological, histopathological, microbiological, radiological, etc.) are also relevant for forensic medicine, though they are not initially encoded. The interpretation of these cases, called “opinion”, consists of a brief report by the veterinary pathologist, based on available necropsy and laboratory results. Cases can be classified into various levels of detail based on the cause of death, the nature of the event leading to the animal's death, and the presence of injuries and/or specific laboratory tests.

The aim of this study is to analyze VFM cases that are non-pre-coded but originate from cases accompanied by an opinion from the archives of the “Istituto Zooprofilattico Sperimentale of Abruzzo and Molise” (IZSAM) to provide an overview of criminal acts against animals in the region, ultimately supporting investigative efforts by judicial authorities tasked with addressing such crimes and to assess the potential for extending this approach to a broader territorial context.

## 2 Materials and methods

### 2.1 Data selection

Necropsy results from livestock, pets, and wildlife, from Abruzzo and Molise regions, performed between 2013 and 2023, were extracted from the IZSAM database. Using internal information systems and filters, it was possible to select, from all the necropsies performed during this period, only those accompanied by a pathologist's “opinion”. Cases pre-coded at the time of sample acceptance for specific topics such as “poisoning” and “wild animals” were also excluded.

### 2.2 Classification of cases

A diagnostic label was assigned to each opinion, by a pathologist specializing in forensic medicine. In case of doubt regarding the classification of certain cases, the pathologist who performed the necropsy was consulted. The cases were then categorized using a three-level hierarchical system. The first level consisted of a macro-category describing the general cause of the animal's death, the second level provided a subcategory specifying the type of event leading to the death, and the third level included notes or details about the circumstances of death, specific injuries, and/or case-specific laboratory tests.

Necropsy opinions of forensic interest were grouped into five macro-categories of criminal events against animals: *neglect, attack, injury, unlawful killing*, and *other*.

Cases labeled under the *neglect* macro-category were further divided into three subcategories: *failure to feed, failure to care*, and *failure to protect*. The *failure to feed* subcategory referred to animals found in a cachectic state without signs of chronic-degenerative or proliferative diseases. The severe and progressive weakening that led to the animal's death was linked to drastic reductions in food or water intake, potentially due to intentional neglect (e.g., confinement), making it a case of malicious intent. The *failure to care* subcategory included animals that died from serious parasitic or infectious diseases, chronic conditions, or trauma, where the caretaker's intervention was absent, as indicated by the medical history. The *failure to protect* subcategory involved cases where animals were not safeguarded from environmental hazards, constituting a form of abandonment.

The *attack* macro-category included cases of animal assaults characterized by bite injuries. It contained three subcategories: *interspecific, intraspecific*, and *predation*. The *interspecific* subcategory included attacks by one species on another, excluding cases categorized as predation, which involved a predator (typically a wild carnivore) attacking its prey.

The *injury* macro-category included cases in which the wounds were caused by inanimate objects. It was divided into subcategories for different types of trauma: *blunt object trauma, compressive object trauma*, and *unspecified trauma*.

The *unlawful killing* macro-category included the following subcategories: *firearm killing, cutting weapon, pointed instrument, trap*, and *mercy killing*.

The *other* macro-category was used to collect forensic veterinary cases (FVCs) that did not fit into the previous categories. These cases often involved autopsies conducted to determine the cause and manner of sudden deaths not attributable to known pathological conditions, potentially suggesting illegal killing. This macro-category has been divided into five subcategories: deaths caused by *physical and environmental agents*, deaths following *post-surgical complications*, deaths of animals seized during *illegal animal trade investigations, crime scene finds*, and *suspected illegal killing*. The *physical and environmental agents* subcategory included cases in which death was caused by environmental factors (e.g., electrocution, plant poisoning). The *post-surgical complications* subcategory included cases in which death occurred as a result of complications arising after surgery or medical treatment, and the necropsy revealed evidence of internal hemorrhage, peritonitis, sepsis, or shock. The *illegal animal trade investigations* subcategory included cases related to the illegal trade in animals, such as cases of fraud involving animal identification or deaths from infectious diseases among animals that have not been vaccinated with mandatory vaccines. The *crime scene finds* subcategory involved samples collected during crime scene inspections for laboratory analysis, often requiring morphological or genetic identification of animal remains found in the environment. The *suspected illegal killing* subcategory involved unearthed carcasses or remains that could not undergo full autopsies, with the investigation relying on visual inspections and laboratory analysis of collected samples.

**Figure 1:**
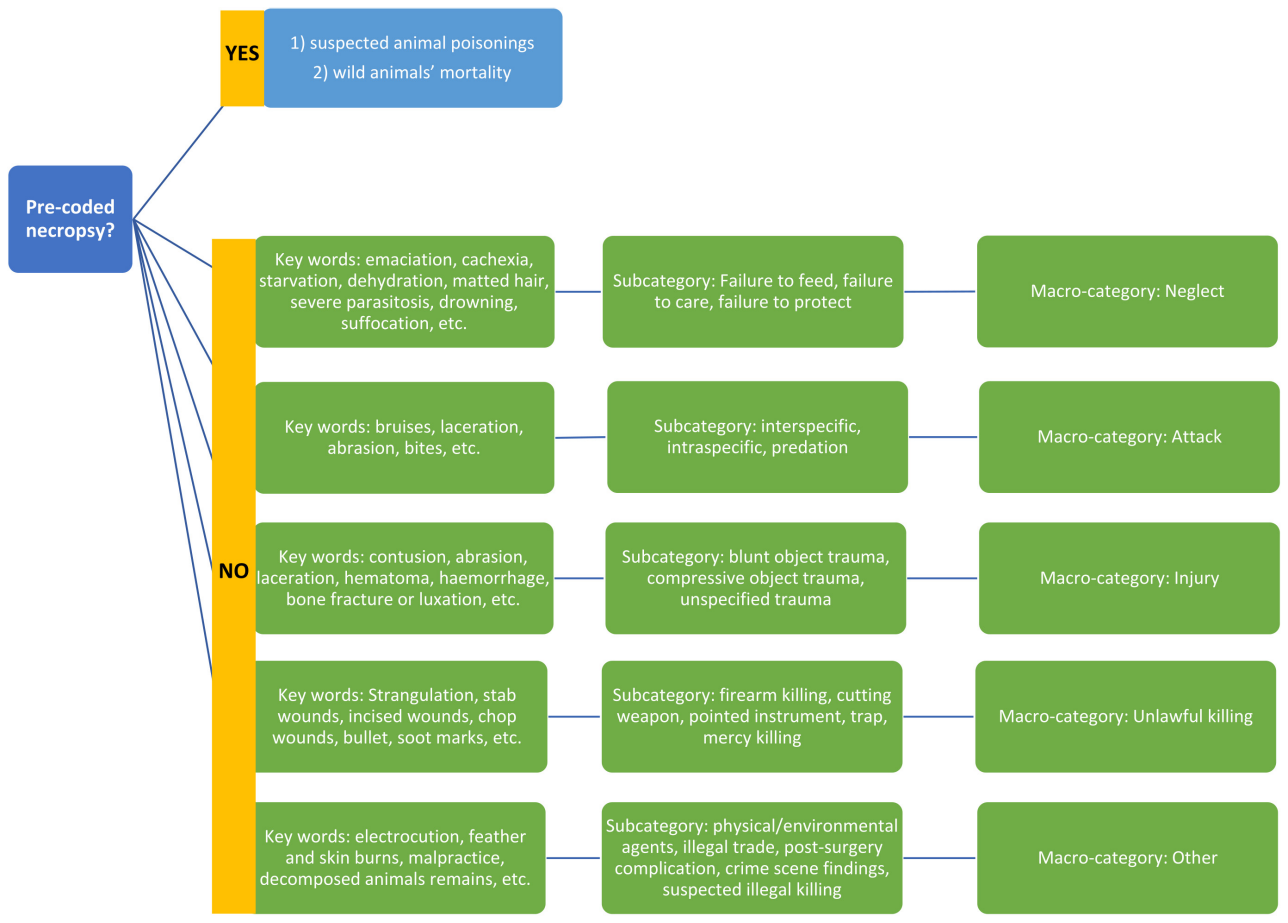
Decisional algorithm. Many keywords are interchangeable and may belong to multiple categories.

The algorithm used during the decisional process for the classification is showed in Figure 1.

## 3 Results

In the period of observation, a total of 1,221 non-coded cases with an opinion were identified in the IZSAM database. Of these, 405 (33.16%) were potential FVCs, 465 (38.08%) were classified as infectious/parasitic diseases, and 351 (28.74%) as organ pathology. The 405 FVC were categorized into five macro-categories and 19 subcategories, as reported in Table 1.

**Table 1:** Classification of VFM cases into macro-categories and subcategories.

Macro-category	Subcategory	Cases *n* (%)
Neglect		57/405 (14.07)
	Failure to feed	7/57 (12.28)
	Failure to care	40/57 (70.17)
	Failure to protect	10/57 (17.54)
Attack		83/405 (20.49)
	Interspecific aggression	16/83 (19.27)
	Intraspecific aggression	32/83 (38.55)
	Predation	35/83 (42.16)
Injury		133/405 (32.84)
	Blunt force injury	72/133 (54.13)
	Pressure injury	25/133 (18.79)
	Injury of unspecified origin	36/133 (27.06)
Unlawful-killing		75/405 (18.51)
	Killing by firearm	63/75 (84.00)
	Killing by cutting weapon	2/75 (2.66)
	Pointed tool	2/75 (2.66)
	Trap	4/75 (5.33)
	Merciful killing	4/75 (5.33)
Other		57/405 (14.07)
	Physical/environment agents	5/57 (8.77)
	Illegal trade	3/57 (5.26)
	Post-surgery complication	12/57 (21.05)
	Crime scene finds	14/57 (24.56)
	Suspected illegal killing	23/57 (40.35)

The third level details (texts of specific cases) were not shown.

**Figure 2:**
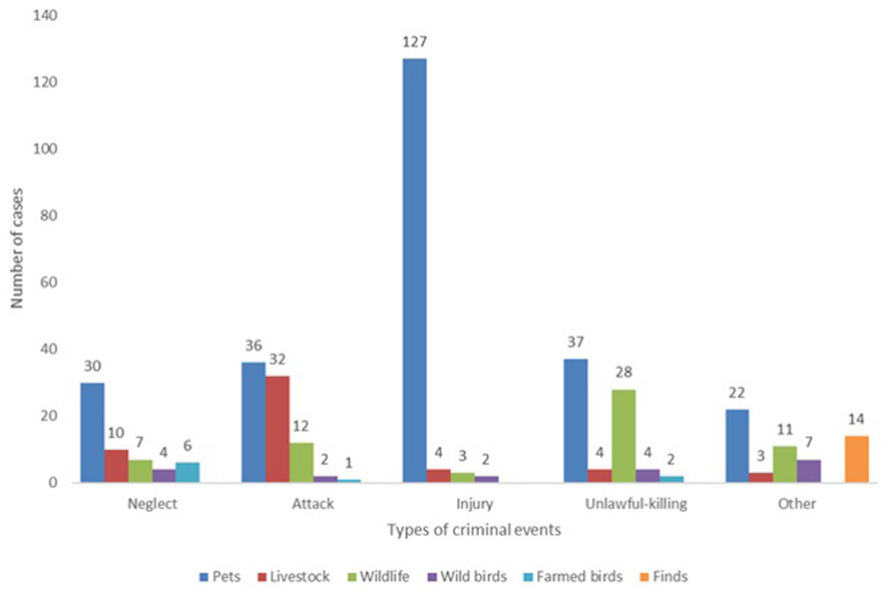
Classification of animals' categories involved in the five types of criminal events.

The classification of the animals involved in the five types of criminal events was as follows: 252 pets (62.22%), 58 wildlife (14.32%), 53 livestock (13.09%), 19 wild birds (4.69%), nine farmed birds (2.22%), and 14 finds (3.46%; Figure 2).

In detail, among the FVCs: 57 cases (14.07%) were classified under *neglect*, 83 (20.49%) under *attack*, 133 (32.84%) under *injury*, 75 (18.51%) under *unlawful killing*, and 57 (14.07%) under *other*.

### 3.1 Neglect

**Figure 3:**
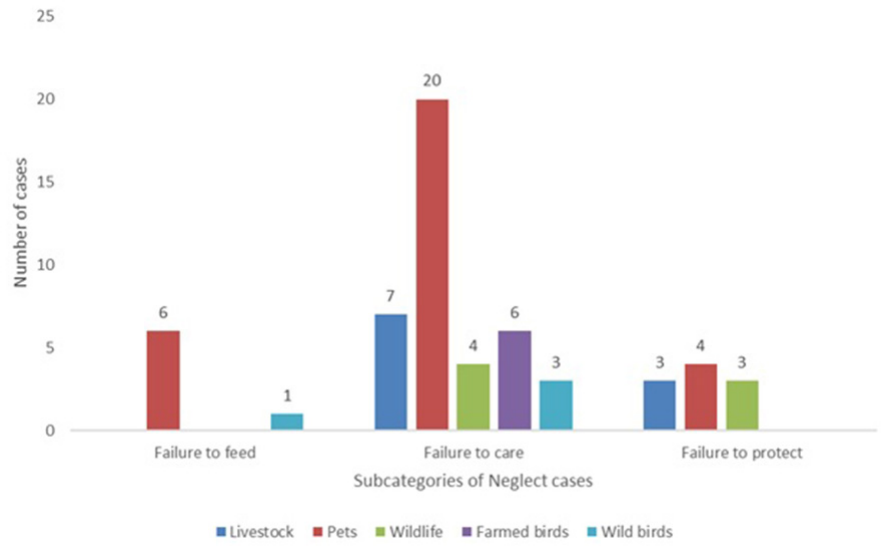
Distribution of neglect cases for different animals' categories divided by subcategories.

Figure 3 shows the details of neglect cases across different animal types, divided by subcategory. The *failure to feed* subcategory included 7/57 cases (12.3%), *failure to care* 40/57 cases (70.2%), and *failure to protect* 10/57 cases (17.5%).

The *failure to protect* subcategory included incidents such as three cases of drowning, two dogs dying from heatstroke during transport, one dog succumbing to frostbite, three cases of chickens asphyxiating due to faulty air conditioning in intensive farming, and one dog died during transport in an airplane's cargo hold. Wild animals in this subcategory included bears that drowned in an uncovered rainwater tank within a protected area, despite an order issued by a forestry authority to install protective fencing. The latter two subcategories are classifiable in a negligent liability profile.

### 3.2 Attack

**Figure 4:**
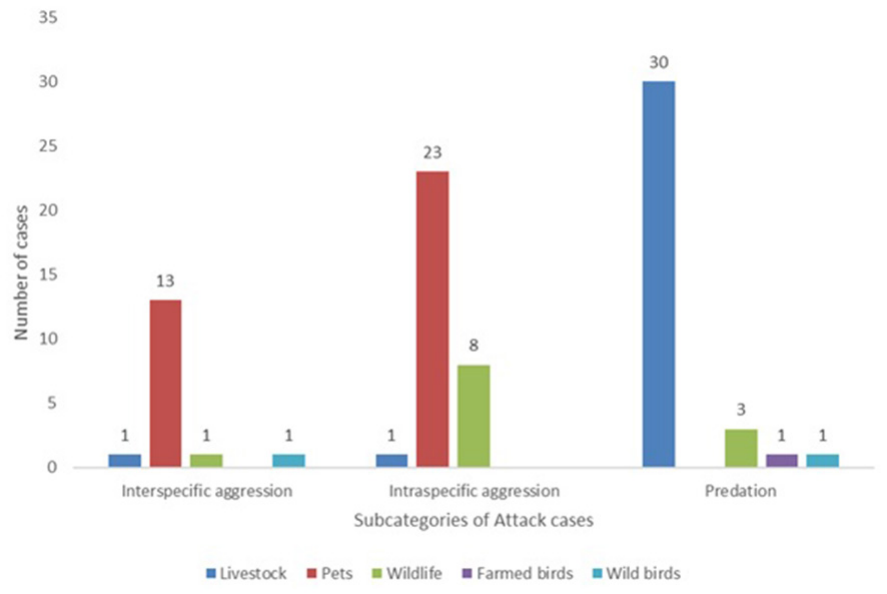
Distribution of attack cases for different animals' categories divided by subcategories.

Figure 4 details the cases based on animal category. The *interspecific* subcategory (16/83, 19.3%) mostly involved pets (13/16). In detail, incidents included eight dog-on-cat attacks, four wolf-on-dog attacks, and one wild boar attacking a dog. The remaining three cases involved dog-chicken, wild boar-wolf, and fox-hawk conflicts. The *intraspecific* subcategory (32/83, 38.5%) involved mostly dog-on-dog attacks (23/32), with one case of overcrowded chickens mutilating each other, and for wild animals, including wolf-on-wolf and bear-on-bear attacks. *Predation* (35/83, 42.2%) was the most common subcategory, pre-dominantly involving farm animals (30/35), specifically 23 cases affecting sheep and goats, five chickens, and two rabbits. Remaining cases (5/35) involved roe deer, wild boar, hare, hawk, and pigeon.

### 3.3 Injury

**Figure 5:**
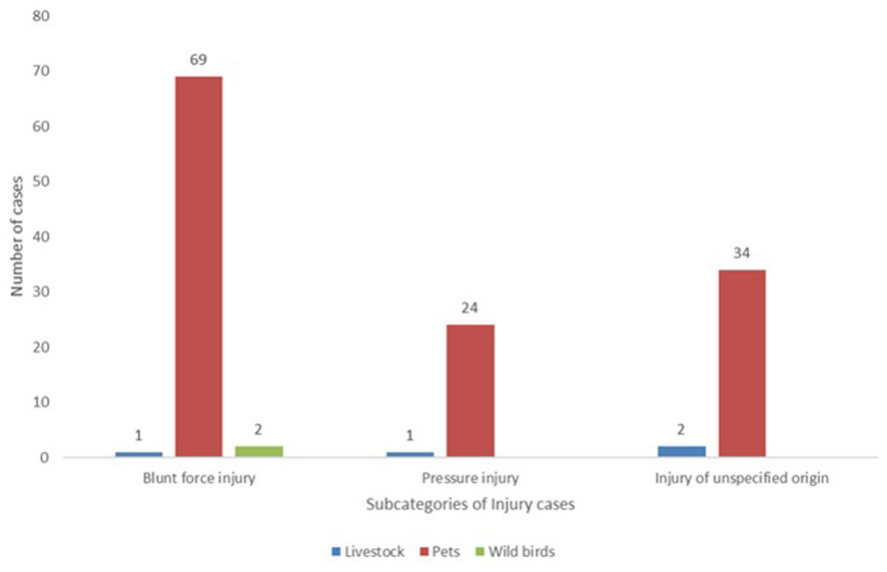
Distribution of injury cases for different animals' categories divided by subcategories.

Figure 5 shows the details of injury cases by animal category. Pets (dogs and cats) accounted for 95.5% of the cases (127/133), while farm animals were involved in four cases (two sheep/goats, one chicken, one turkey), and wild birds in two cases (one starling, one mallard).

### 3.4 Unlawful killing

**Figure 6:**
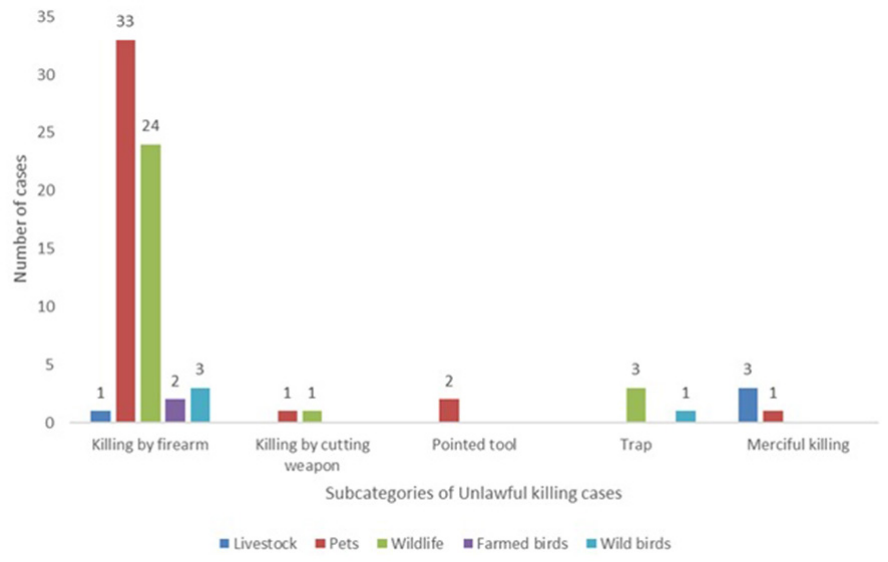
Distribution of unlawful killing cases for different animals' categories divided by subcategories.

Figure 6 shows the details of unlawful killing cases divided by animal category involved. *Killing by firearm* was the most represented subcategory (63/75, 84%) which mainly involved pets (33/63) and wild animals (24/63). Pets included 23 dogs and 10 cats. *Killing by cutting weapon* included two cases that involved a dog and a wild boar. The latter, specifically, died following penetrating body trauma (use of a crossbow) and the profile of responsibility was delineated based on the period of the year in which the event occurred, not falling within the authorized hunting season. *Pointed instrument* included two cases involving a dog and a colony of cats. *Trap* grouped three cases of use of a self-tightening noose against two wolves and a fox, and the use of a plastic net to capture wild birds. *Mercy killing* involved unacceptable methods of killing suffering animals. In two cases, cattle and a horse were killed with a captive bolt, a sheep with a sharp weapon, and a dog with a hammer.

### 3.5 Other

**Figure 7:**
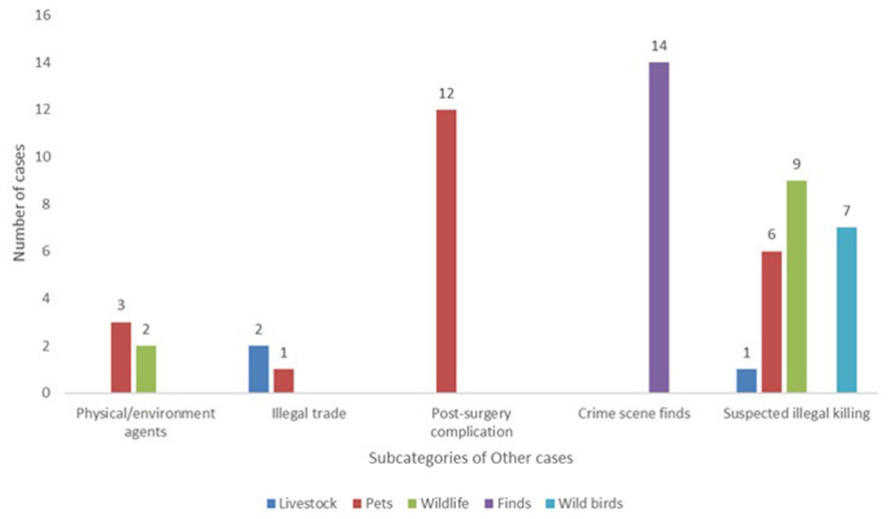
Distribution of other cases for different animals' categories divided by subcategories.

Figure 7 provides details of the *other* cases by animal category. The *physical/environmental agents* subcategory (5/57, 8.8%) included two electrocution cases (hares and chamois), one dog poisoned by palm seeds, and two animals (dog and cat) that died from necrosis caused by processionary caterpillar larvae. The *illegal trade* subcategory included two cases of ear tag replacement in sheep and one case of mortality by viral gastroenteritis in undocumented purebred puppies seized during illegal transport. The *post-surgery complication* subcategory (12/57, 21.0%) involved pets that died from various causes following surgery. These cases often arose from disputes between owners and veterinarians, where FVM laboratories were used to assess potential professional liability. The *crime scene findings* subcategory (14/57, 24.5%) included carcass remains or pathological materials sent for inspection or species identification. The *suspected illegal killing* subcategory (23/57, 40.3%) involved cases where autopsies could not be performed, but inspections and laboratory testing were conducted to investigate the cause of death.

## 4 Discussion

From the analysis of post-mortem reports with “opinions”, the main cause of mortality in potentially forensic cases varied by animal type. For pets, the primary cause was *injury* (50.4%), for livestock it was *attack* (60.4%), and for wildlife, *unlawful killing* (40%). Poisoning cases were excluded from this study as they are pre-classified. Similarly, wild animals' deaths caused by vehicular collisions were not considered, as they are automatically coded under “wild animals” and excluded from this study. Therefore, the percentage of wildlife included in the analysis derived from those cases that were not pre-classified, but were subjected to a necropsy requiring opinion.

The majority of deaths classified in the macro-category *injury* were due to vehicular collisions involving animals other than wild animals. These findings align with previous research by Marchetti et al. (7), which also identified *injury* as the leading cause of death. The types of criminal events described in this study also correlate with findings by Ottinger et al. (2), who surveyed veterinary pathology laboratories across Europe. Ottinger's study (2) identified poisoning (score 34), abandonment (9), cruelty (10), insurance claims (11), unlawful killing (12), and poaching (7) as the most common forensic veterinary issues. While Ottinger's data (2) were not expressed as percentages, it still shows a similar pattern to the results of this study, with the exception of “cruelty,” which appeared only once in our research in a case involving the mistreatment of a colony of five cats.

Insurance-related cases can often be linked to the *injury* category, particularly in instances of vehicular collisions. However, the *injury* category also includes cases of animal beatings, which may involve intentional cruelty, leading to intentional liability.

In this context, postmortem diagnostic imaging techniques such as X-rays, computed tomography (CT) scans, and magnetic resonance imaging (MRIs) can be very useful in determining whether a death was accidental or not, as these techniques are objective and can strengthen the case's legal standing in court (13). Autopsy imaging (Ai) may reveal fractures, subcutaneous emphysema, pneumothorax, pneumoperitoneum, diaphragmatic hernias, and abdominal ruptures. These images provide the pathologist with preliminary information regarding the location of lesions prior to the necropsy, thereby guiding the pathologist on which areas to focus on most closely and where to take samples before the post-mortem examination. Furthermore, gases, which can easily escape during the necropsy, are easily detectable on CT scans (13). They can also help determine the characteristics of the bullets and their trajectory in cases of gunshot wounds (14). The use of Ai can be helpful in the field of VFM, although its application may be limited by financial constraints, the availability of facilities, and human resources (13).

Aleksic Radojkovic et al. (15) reported that of 338 dogs subjected to necropsy, 2.1% (7/338) of deaths were attributable to motor vehicle accidents, while 9.3% (5/54) occurred in cats. The finding of a lower percentage in this work in Serbia was attributed by the authors to the fact that animals that die following these accidents are often not subjected to necropsy, or these cases may have been analyzed separately and classified as accidental injuries. A study conducted in Spain between 2014 and 2019, which examined causes of death in companion animals, reported that 43.64% of dogs and 21.95% of cats died from blunt force trauma (12). A more recent Spanish study covering 2020–2024 specifically investigated 53 cats with suspected abuse and found that 32.07% died from blunt force trauma (16). A study conducted in Portugal (17), which evaluated cases of suspected crime against pets, found that 31% and 100% of the deaths among the cases in which post-mortem analyses was compatible with violent death, respectively of dogs and cats, were due to blunt force trauma. However, the number of cats involved was low, probably due to the particular behavior of cats, which, when injured or scared, tend to hide and are more difficult to detect. A retrospective study of cases of dog and cat abuse in the city of São Paulo also found that mechanical force was the second most common type of abuse, accounting for 21% of cases in dogs and 24% in cats. However, according to these authors, because trauma can be caused by people living with the animal, these cases often go unreported, which may mask a larger caseload than revealed (18). The high number of cases reported in the present study reveals a high level of public awareness and sensitivity toward animal welfare and good work by public veterinary health services.

According to technical reports on cases pre-coded as “wild animals” by IZSAM, vehicular collisions were the primary cause of death, followed by poisoning (Data not shown). Data that echoes Marchetti et al. (7), who reported vehicular collisions as the cause of death in 65.8% of wildlife cases. The remaining deaths were attributed to animal aggression (16.8%), poisoning (3%), intentional or accidental, and firearm shots (1.8%). Although Marchetti's (7) study examines a different number of cases and uses different classifications of injuries, the wildlife mortality models are comparable.

The study of wildlife mortality highlights the critical need to protect vulnerable species, especially in regions like Abruzzo, where 36% of the territory is protected. This region harbors significant biodiversity, including species under national and international protection, such as the Marsican brown bear (*Ursus arctos marsicanus*), the Apennine chamois (*Rupicapra pyrenaica ornata*), and the wolf (*Canis lupus*). However, increasing wildlife populations and their expanded habitats have led to greater overlap with human activities, heightening risks to biodiversity, such as the spread of infectious diseases and the impact of human infrastructure on wildlife. In fact, according to environmentalists and environmental planners, roads and traffic can reduce or even eliminate wildlife populations (19).

To mitigate these risks, there is a pressing need to design transport infrastructure—highways, roads, railways—that minimizes environmental damage, such as habitat fragmentation, which increases wildlife mortality from vehicular collisions. “Road ecology,” a field that integrates engineering and wildlife biology, addresses these ecological impacts (20). Currently, Italy lacks a national system for monitoring road-related wildlife deaths, relying on local studies. A systematic approach could help identify high-risk road sections and implement measures such as visual deterrents, fencing, and wildlife crossings.

For pets, cases other than *injury* were distributed across the forensic macro-categories *neglect* (11.9%), *attack* (14.3%), and *unlawful killing* (14.7%), with 8.7% falling into other causes. A neglected animal is defined as an animal deprived of one or more animal's basic needs, namely food, water, shelter, or necessary veterinary care (6, 11). Neglect is suspected when pica, dehydration, severe parasitic infestations, and obvious lack of medical care are observed. Many studies report high rates of neglect among the forensic cases analyzed. A retrospective United States study found neglect in 52.1% of necropsy cases suspected of animal abuse (21, 22). Within the neglect macro-category, the subcategory *failure to care* (70.2%), which include the majority of cases of the *neglect* macro-category, like the subcategory *failure to protect* (17.5%), often involve negligence, leading to a careless liability profile. Conversely, *failure to feed* (12.3%) was typically the result of intentional abandonment (e.g., confinement) and therefore classified as an intentional act. In this case, the primary pathological finding is malnutrition, characterized by a reduction in subcutaneous fat, with wrinkled skin due to dehydration, muscle atrophy, and a reduction in visceral fat. However, it is always necessary to distinguish whether malnutrition was caused by an inadequate or unbalanced diet, or by the body's inability to properly digest or absorb food (6).

In the *attack* macro-category, responsibility is usually linked to negligence, such as failure by pet owners to control their animals in cases of intra- or interspecific aggression, or inadequate control measures by authorities in the case of livestock predation by wild carnivores. A 2018 Australian retrospective study reported that, over the course of 1 year, 2.7% (418 dogs) of the total dog population was attacked by another dog (intraspecific aggression), while 1.1% (41 cats) of the cat population was attacked by a dog (interspecific aggression) (10). A study conducted in Taiwan between 2019 and 2020 on fatal injuries caused by dogs in free ranging cats reported that 53.6% had blunt-force trauma, and of these, 34.8% had animal bite injuries (23). Due to their intense social interactions and their close adaptation to living alongside humans in confined areas, dogs are particularly vulnerable to behavioral disturbances and related forms of abuse (24).

The *unlawful killing* macro-category covers intentional acts against both domestic and wild animals, with the most common subcategory being *killing by firearm* (84%). For wild animals, such killings often fall under poaching, especially when they involve species not authorized for hunting or occur outside the hunting season. In Abruzzo, wild boar, hare, and fox are the only mammals subject to regulated hunting, meaning that the intentional killing of other species constitutes poaching. Even animals typically authorized for hunting can be poached if killed outside the legal season.

Poaching, a growing issue in Italy, particularly due to the expanding populations of wild ungulates and the increase in livestock predation by carnivores, is difficult to quantify but has significant consequences for biodiversity. The European Commission has even raised concerns about the high rates of illegal killings in Italy (25). Our study identified 24 cases of poaching involving firearms (targeting species like roe deer, red deer, wild boar, otter, wolf, and fox), one case involving a crossbow (wild boar), and four cases involving traps (wolf, fox, and birds). Other literature (7) reports similar cases involving firearms, snares, or drowning.

Finally, the *other* macro-category includes cases of death due to *physical/environmental agents* (8.8%), which are often linked to negligence. This subcategory includes cases of animals that have died from electrocution. This issue is generally associated with bird deaths, which in the United States are estimated to affect between 0.9 and 11.6 million birds each year. The post-mortem examination in these suspected cases must be thorough because the signs of an electrocution injury can be subtle and easily overlooked if only a superficial assessment is performed; therefore, this possibility must always be considered in cases of birds found dead near high-voltage power lines (26). The main lesions observed during necropsy are burns on the feathers and skin, but these may also be small or covered by blood or dirt, making them difficult to detect (26). Other lesions typically found include fractures, as well as cardiac, vascular, and neurological damage. In wild animals, skin burns and destruction of underlying tissues such as muscles, tendons, and bones may be observed (6). The pathological findings may differ when animals are struck by lightning; in such cases, the electrical current passes through the body very rapidly and primarily affects the outer parts of the body, with linear burns typically observed on the medial aspect of the limbs.

In the *illegal trade* subcategory, genetic investigations have often been used, which in recent years have been developed with cutting-edge techniques also in animals, not only in pets, but also in livestock. In fact, various cattle (*Bos Taurus*) STR loci of cattle have been deposited (9) and Lorenzini et al. reported a case report that used 16 STR loci to link a mouflon carcass to items sized from a vehicle (27).

Cases of *post-surgery complications* (21%) frequently involve potential veterinarian liability, requiring impartial investigation by forensic veterinary laboratories.

## 5 Conclusions

The process of data extraction, based on assigning diagnostic labels to veterinary pathologists' opinions, enabled the systematic classification of selected cases into well-defined categories of potential criminal acts against animals. This method provided valuable insights into the extent of these criminal events in the Abruzzo and Molise regions, offering a useful tool for supporting forensic investigations.

This method is particularly suited to the context of IZSAM, where forensic cases are often non-pre-coded but rather identified during routine diagnostic activities. This study integrates the landscape of topics of veterinary forensic medicine helping to create a more comprehensive archive of all forensic or potentially forensic cases in the regional territories. However, it is important to note that to obtain a complete picture of mortality rates, these data must be combined with pre-coded cases related to suspected poisoning and wildlife roadkill. The authors hypothesize that this type of approach could also be used by the network of IIZZSS, distributed throughout Italy, as the procedures for sample acceptance and reporting are similar.

In this way, the IIZZSS archives can serve as a comprehensive observatory for FVM-related issues, given that they perform the majority of postmortem examinations in their jurisdiction, offering valuable insights into criminal activities involving animals within their jurisdiction. Moreover, categorizing cases into major forensic categories enables the compilation of statistics and a better understanding of the prevalence of different criminal acts, ultimately supporting the prevention and the investigative work of authorities tasked with addressing these crimes.

## Data Availability

The datasets presented in this study are not publicly available due to restrictions imposed by a governmental health institution. Access to the data may be granted upon reasonable request, subject to authorization by the competent authority. Requests to access the datasets should be directed to a.petrini@izs.it.
